# Leveraging Prior Knowledge to Recover Characteristic Immune Regulatory Motifs in Gulf War Illness

**DOI:** 10.3389/fphys.2020.00358

**Published:** 2020-04-28

**Authors:** Saurabh Vashishtha, Gordon Broderick, Travis J. A. Craddock, Zachary M. Barnes, Fanny Collado, Elizabeth G. Balbin, Mary Ann Fletcher, Nancy G. Klimas

**Affiliations:** ^1^Department of Medicine, University of Alberta, Edmonton, AB, Canada; ^2^Center for Clinical Systems Biology, Rochester General Hospital, Rochester, NY, United States; ^3^Department of Biomedical Engineering, Kate Gleason College of Engineering, Rochester Institute of Technology, Rochester, NY, United States; ^4^Institute for Neuro-Immune Medicine, Nova Southeastern University, Fort Lauderdale, FL, United States; ^5^Departments of Psychology & Neuroscience, Computer Science and Clinical Immunology, Nova Southeastern University, Fort Lauderdale, FL, United States; ^6^Diabetes Research Institute, University of Miami, Miami, FL, United States; ^7^Miami Veterans Affairs Medical Center, Miami, FL, United States

**Keywords:** Gulf War Illness, cytokines, immune signaling, Th1, Th17, network biology, prior knowledge, simulation

## Abstract

Potentially linked to the basic physiology of stress response, Gulf War Illness (GWI) is a debilitating condition presenting with complex immune, endocrine and neurological symptoms. Here we interrogate the immune response to physiological stress by measuring 16 blood-borne immune markers at 8 time points before, during and after maximum exercise challenge in *n* = 12 GWI veterans and *n* = 11 healthy veteran controls deployed to the same theater. Immune markers were combined into functional sets and the dynamics of their joint expression described as classical rate equations. These empirical networks were further informed structurally by projection onto prior knowledge networks mined from the literature. Of the 49 literature-informed immune signaling interactions, 21 were found active in the combined exercise response data. However, only 4 signals were common to both subject groups while 7 were uniquely active in GWI and 10 uniquely active in healthy veterans. Feedforward mediation of IL-23 and IL-17 by IL-6 and IL-10 emerged as distinguishing control elements that were characteristically active in GWI versus healthy subjects. Simulated restructuring of the regulatory circuitry in GWI as a result of applying an IL-6 receptor antagonist in combination with either a Th1 (IL-2, IFNγ, and TNFα) or IL-23 receptor antagonist predicted a partial rescue of immune response elements previously associated with illness severity. Overall, results suggest that pharmacologically altering the topology of the immune response circuitry identified as active in GWI can inform on strategies that while not curative, may nonetheless deliver a reduction in symptom burden. A lasting and more complete remission in GWI may therefore require manipulation of a broader physiology, namely one that includes endocrine oversight of immune function.

## Introduction

Gulf War Illness (GWI) is a poorly understood illness (Wolfe et al., 1998) associated with deployment to the Persian Gulf between 1990 and 1991. Varying significantly according to a veteran’s assigned location in theater (Steele et al., 2012), GWI presents as a complex constellation of symptoms that include fatigue, widespread pain, cognitive and memory problems, skin rashes, gastrointestinal and respiratory difficulties (White et al., 2016). Affecting several of the body’s principal regulatory systems (Unwin et al., 1999; Bourdette et al., 2001; Kang et al., 2003) GWI is now thought to involve a neuroinflammatory pathology arising from exposures to a range of organophosphates including pyridostigmine, DEET and sarin exacerbated by environmental stressors in theater (Amourette et al., 2009; Barbier et al., 2009; O’Callaghan and Miller, 2019). Among these, our work and the work of others suggest alterations to the hypothalamic-pituitary-adrenal (HPA) response to challenge (Golier et al., 2006, 2007; Rice et al., 2016; Craddock et al., 2014) and that such alterations may become persistent and stable dysregulations (Rice et al., 2016). Exercise has been used as a minimally invasive means of interrogating HPA axis response (Duclos and Tabarin, 2016), one that is especially appropriate given that a chief presenting symptom of GWI is debilitating fatigue. Though data suggest that peak exercise capacity is comparable (Nagelkirk et al., 2003), these individuals report higher fatigue (Cook et al., 2003) and differ significantly from healthy control subjects in their ability to recover from these challenges. More recently, Rayhan et al. (2013a) have shown that these differences in recovery from exercise may support the identification of GWI subgroups with significant differences in autonomic response and distinct cognitive vs. physical constructs of Chalder fatigue profile (Chalder et al., 1993). Exercise induced exacerbation of symptoms or post-exertional malaise (PEM) (McManimen et al., 2016) has also emerged as a distinguishing feature in a sister illness myalgic encephalomyelitis/chronic fatigue syndrome (ME/CFS) (Snell et al., 2013) with these individuals achieving significantly lower values for oxygen consumption and workload at peak effort and at the anaerobic threshold when re-assessed 24 h after an initial maximal exercise challenge. Our own pilot work has shown that this altered capacity for recovery from exercise also manifests as distinct trajectories in immune marker co-expression (Broderick et al., 2012) and that these illness-specific alterations differ between men and women (Smylie et al., 2013). Signaling patterns that emerge in response to exercise were structurally different in GWI with the latter drawing on a larger network of alternate signaling patterns in an effort to respond adequately to challenge (Broderick et al., 2011). Moreover, these differences were linked to changes in symptom severity, extending through multiple layers of biology from altered patterns of gene expression (Whistler et al., 2009) along select signaling pathways (Broderick et al., 2013). Not unexpectedly, more recent work has shown this also extends to altered cell metabolism (Koslik et al., 2014), an experimental observation further validated with *in silico* simulations of mitochondrial function (Lengert and Drossel, 2015).

While this earlier work by our group supported the association of symptom clusters with characteristic patterns of immune marker co-expression, it was based on samples collected prior to exercise, at peak effort and at 4 hours post-exercise. As a result, the experimental sampling frequency was insufficient to support the identification of classical rate equations models (Vashishtha et al., 2015) that in turn might provide additional insight into the causal mechanisms driving altered immune signaling in GWI. The objective of the present work is to discover such causal mechanisms that might become characteristically activated during exercise in GWI as well as elements of immune regulation that might be conspicuously absent. Toward this we have extended sampling to include 8 blood draws collected prior to, during and up to 4 h after peak exercise in *n* = 12 veterans with GWI and *n* = 11 healthy control veterans (HC). In an effort to cast this data in the context of *a priori* knowledge, we apply as a mechanistic scaffold an extension of a literature-based model of immune signaling (Fritsch et al., 2013) previously reported by our group. We group individual cytokine and chemokine measurements into the functional sets reported by Folcik et al. (2007, 2011) and apply a rate equation framework which leverages the basic topological features of biological networks to infer regulatory control actions rather than rely on a more conventional structurally naïve fit to data (Vashishtha et al., 2015). Candidate causal relationships inferred from the data are then projected onto documented signaling mechanisms extracted from the literature. Results of this analysis again suggest that immune response to exercise in GWI veterans draws on a set of known immune signaling mechanisms that differs significantly from the signaling patterns expressed in healthy veterans. Many of these differences involved mechanisms mediating the coordinated activity of innate immune response with the Th1 and Th17 adaptive immune axes. Consistent with earlier exploratory work by our group, simulated interventions directed at disrupting these abhorrent regulatory motifs resulted in only partial rescue suggesting that lasting remission in GWI may require therapeutic modulation of a broader physiology, namely one that includes endocrine oversight of immune function (Craddock et al., 2015).

## Materials and Methods

### Cohort Recruitment

A subset of *n* = 12 GWI subjects and *n* = 12 healthy control (HC) but sedentary Gulf War era veterans were recruited from a larger ongoing study at the Miami Veterans Administration Medical Center. Subjects were male and ranged in age between 40 and 60, and of comparable body mass index (BMI), ethnicity and duration of illness. Inclusion criteria was derived from Fukuda et al. (1998) and consisted in identifying veterans deployed to the theater of operations between August 8, 1990 and July 31, 1991, with one or more symptoms present after 6 months from at least 2 of the following: fatigue; mood and cognitive complaints; and musculoskeletal complaints. Subjects were in good health prior to 1990, and had no current exclusionary diagnoses (Reeves et al., 2003). Medications that could have impacted immune function were excluded. Use of the Fukuda definition in GWS is supported by Collins et al. (2002). Healthy control subjects were recruited from the veteran population, and the local National Guard units to adjust for military training and vaccination protocols. They were self-defined as sedentary, and were matched to GWI by age, gender, race/ethnicity and BMI as closely as possible. Summary results of the included subset of subject demographics and exercise performance are listed in Table 1. Additional details about recruitment inclusion and exclusion criteria for this cohort can be found in Broderick et al. (2013). Subjects are further described in greater detail with regard to symptom burden measures in Table 1. These measures include the Medical Outcomes Study 36-item short-form survey (SF-36) (Ware and Sherbourne, 1992) assessing health-related quality of life, the Multidimensional Fatigue Inventory (MFI) (Smets et al., 1995), a 20-item self-report instrument designed to measure fatigue, the Pittsburgh Sleep Quality Index (PSQI) (Buysse et al., 1989) and the Davidson Trauma Scale (DTS) (Davidson et al., 1997) and instrument designed to assess symptoms of post-traumatic stress disorder (PTSD).

**TABLE 1:** Summary of demographic variables and exercise performance.

Demographic variable	HC	GWI
Subjects	12	12
Race		
*Caucasian 0*	4	2
*African American 1*	6	4
*Asian 2*	2	6
Age (years)	47.1 (1.3)	45.0 (1.2)
Body mass index (BMI)	30.1 (1.5)	33.4 (1.6)
Time to VO_2_ max (min)	9.8 (1.1)	6.7 (1.6)
VO2 max (ml/min/kg)	25.4 (1.8)	21.0 (2.2)
**SF36 Health Survey (range 0–100, 100 (optimal**)		
Vitality	73.3 (4.0)	53.8 (5.9)
Phys function	80.8 (5.0)	43.8 (9.2)
Physical limit	77.1 (10.9)	14.6 (8.9)
Emotional limit	86.1 (7.7)	25.0 (11.7)
Emotional wellness	58.3 (1.3)	44.3 (4.1)
Social function	82.3 (5.2)	28.0 (8.9)
Pain	64.6 (6.7)	34.2 (7.7)
General	60.4 (3.8)	37.1 (4.2)
**Multidimensional fatigue inventory (MFI**)		
(subscale 0–100, 100 (fatigued)		
General fatigue	34.1 (6.7)	71.7 (6.6)
Physical fatigue	25.8 (7.3)	68.0 (4.8)
Mental fatigue	36.8 (8.3)	80.9 (5.2)
Reduced activity	28.5 (7.0)	62.1 (6.2)
Red motivation	22.0 (5.2)	56.2 (6.6)
PSQI score	7.4 (1.7)	14.1 (1.2)
(0 = No difficulty; 21 = severe difficulty)		
**Davidson trauma scale (TS)**		
(total score 0 – 136, 136 severe)		
DTS total	29.0 (8.9)	88.8 (8.7)
Intrusiveness	9.1 (3.1)	26.6 (2.6)
Avoidance/Numbness	9.3 (3.4)	33.9 (4.1)
Hyperarousal	11.4 (3.7)	30.8 (2.0)

*Demographic variables such as Race, average age, BMI and exercise performance VO2 max levels and average time to VO2 max, as well as average symptom burden measures with their respective standard errors () for Healthy control (HC) and GWI.*

### Ethics Statement

All subjects signed an informed consent approved by the Institutional Review Board of the University of Miami and the Miami Veterans Affairs Medical Center. Ethics review and approval for data analysis was also obtained by the IRB of the University of Alberta.

### Graded eXercise Test

All subjects included in the study (HC and GWI) underwent a standard maximal Graded eXercise Test (GXT) to stimulate immune response. A Vmax Spectra 29c Cardiopulmonary Exercise Testing Instrument, Sensor-Medics Ergoline 800 fully automated cycle ergometer, and SensorMedics Marquette MAX 1 Stress ECG (GE Healthcare, Chicago, IL) were used during the GXT. Following the McArdle protocol (McArdle et al., 2007), subjects pedaled at an initial output of 60W for 2 min, followed by an increase of 30W every 2 min until the subject reached: (1) a plateau in maximal oxygen consumption (VO2); (2) a respiratory exchange ratio > 1.15; or (3) the subject stopped the test. A total of 8 blood draws were conducted on each subject. First blood draw (T0) was conducted prior to exercise following a 30-min rest. Second and third blood draws were conducted ∼3–5 min. after starting the exercise test (T0 + 3) and upon reaching peak effort (VO2 max) (T1) respectively, followed by blood draws at 10, 20, 30, 60 min., and 4 h after VO2 max (T1 + 10, T1 + 20, T1 + 30, T1 + 60, and T2). All control subjects were screened as sedentary upon recruitment on the basis of their response to a questionnaire (Hurwitz et al., 2009). Analysis in this same cohort (Smylie et al., 2013) of the weight-adjusted maximum VO2 measured in ml/min/kg indicated a decline in the average maximum VO2 achievable with healthy controls performing best (*p* = 0.04). In light of this finding we suggest that results presented here be interpreted as immune response at maximum perceived exertion but not necessarily at equivalent exercise intensity. We consider reduced exercise capacity to be another symptom of GWI. The characteristic immune response patterns measured at maximum perceived exertion capture this implicitly. Exercise performance obtained in this cohort for subjects in each group are reported in Table 1.

### Cytokine Profiling

Plasma was separated from each sample within 2 h of collection and stored at −80°C until assayed. Concentration levels of 16 cytokines were measured in plasma using Quansys reagents and 96 well plates based chemiluminescent imaging instrument (Quansys Biosciences, Logan, Utah). The Q-Plex^TM^ Human Cytokine- Screen (16-plex) is a quantitative enzyme-linked immunoabsorbent assay (ELISA), where 16 distinct capture antibodies have been absorbed to each well of a 96-well plate in a defined array. The range of the standard curves and exposure time were adjusted previously to provide reliable comparisons between subject groups in this illness population at both low and high cytokine concentrations in plasma. Quadruplicate determinations were made, i.e., each sample was run in duplicate in two separate assays. The standard sample concentrations used to establish second order polynomial calibration curves for each cytokine as well as the detection limits for this assay have been described in detail in previous work by our group (Broderick et al., 2010). In brief, these support an average coefficient of variability (CV) of 0.20 for inter-assay comparisons and a value of 0.09 for intra-assay repeatability. Raw values for cytokine concentration levels (pg/ml) are reported in Supplementary Table S1.

### Quantitative Analysis

#### Statistical Evaluation of Cytokine Data

Prior to analysis, the raw cytokine concentration data was filtered and normalized. All the cytokine levels that were undetectable by ELISA (zeros) were replaced by the minimum concentration level of that particular cytokine observed across all the subjects (HC and GWI). The raw cytokine data was linearly interpolated across the entire time course using the minimum time interval (i.e. ∼3 min) to provide equally spaced sample estimates. Further, interpolated data was log2 transformed and normalized for every cytokine across both groups by subtracting the average log2 transformed cytokine levels at rest (T0) in the HC group for each cytokine. This normalized log2 transformed data is finally converted to fold change by calculating the log2 normalized cytokine concentration exponent of 2. Summary statistics of the filtered and normalized cytokine concentration levels (pg/ml) are reported in Supplementary Table S2. It is important to note that these summary statistics are applied to measurements sharing the same target sampling time. As differences in alignment of individual time courses arise as a result of the exercise capacity and response kinetics of each individual, important response features may be easily obscured. This is only further complicated in the case of more complex oscillatory behavior such as that observed in this exercise response data as reported previously by our group (Lyman et al., 2019). For this reason, we have focused the analysis on this work on the recovery of network motifs and response mechanisms active in each individual subject’s exercise challenge and common across individuals within each group. Though indicative of overall trends, the statistics presented in Supplementary Table S1 were computed primarily as an indication of the range of values recorded. Accordingly, we identified an outlier in the healthy control subjects with out-of-range expression levels in several cytokines, namely IL-2, IL-6 and IL-13. This subject was removed from further study leaving *n* = 11 healthy control subjects (Supplementary Figure S1).

Differences in the time course response to exercise for individual cytokines separating one subject from the next were characterized using the SMETS (Semi Metric Ensemble Time Series) measure. This measure was developed for the comparison of multiple time series of arbitrary and unequal length (Tapinos and Mendes, 2013) and is well suited to the misalignments in sampling time and the varying duration in exercise response observed in this work as a result of differences in fitness level and illness severity. In order to generate distribution statistics for this measure of divergence in trajectory we combined a leave-one-out cross-validation strategy with a standard bootstrapping. More precisely, 11 subsets *n* = 10 of 11 (leave-one -out in HC) subject time series were randomly sampled without replacement and were compared with another equal-sized group of 11 subsets of *n* = 10 of 12 (leave-two-out in GWI) subject time series also randomly sub-sampled without replacement. This was done for each of the 16 cytokines. Intra-group and inter-group SMETS values were calculated by comparing each of the 10 subsampled time series from one group with each other as well as with each of the 10 subsampled time series from the opposing group. This is repeated 11 times. Differences in the resulting distributions of intra and inter-group SMETS values were tested for significance using the standard two-sample *t* test and the Wilcoxon ranksum test (Supplementary Table S3).

#### Aggregating Cytokines Into Functional Sets

In previous work, our group updated and further developed the network of documented immune signaling interactions used by the agent-based Basic Immune Simulator (Folcik et al., 2007) to create an augmented model of innate and adaptive immune cell signaling (Fritsch et al., 2013). In this model, various adaptive immune cell subsets were aggregated into functional groups (namely, T helper cell populations Th1, Th2, Th17, and cytotoxic T lymphocytes CTL) as were sub-populations of innate immune cells (natural killer cells NK and dendritic cells DC). In much the same way individual cytokines were grouped based on the predominant cell population of origin and mode of action (see Table 2). For example, cytokines released primarily by monocytes (DCs) were grouped into a monokine (MK) group whereas cytokines released by lymphocytes such as NKs, Th1, Th2, and CTLs are grouped into a cytokine group (CK). These groups were further subdivided into monokine MK1 and cytokine CK1 forming the pro-inflammatory cytokine functional sub-groups, with MK2 and CK2 comprising the corresponding anti-inflammatory sets. The remaining cytokine groups were composed of individual cytokines (e.g., MK15 contains only IL-15).

**TABLE 2:** Aggregated Cytokine groupings.

Node ID	Group	Cytokines
1.	MK1A	IL-1(α, IL-1(β, IL-8 and IL-12
2.	MK1B	IL-1(α, IL-1(β, IL-8 and IL-12
3.	MK2	IL-10
4.	MK6	IL-6
5.	MK15	IL-15
6.	MK23	IL-23
7.	CK1	IL-2, IFN(γ, TNF(α and TNF(β
8.	CK2	IL-4, IL-5 and IL-13
9.	CK17	IL-17

*Resulting aggregated variables used in the extracted cytokine network from the immune signaling model reported in Fritsch et al. (2013). MK1A and MK1B represent the first and second principal components respectively obtained from individual cytokines.*

In order to take advantage of this documented signaling network it was necessary to aggregate the individual cytokines measured experimentally in this work into their corresponding functional sets as defined in Fritsch et al. (2013). In cases where the functional sets contained multiple cytokines, such as MK1, CK1 and CK2, the activation level for the aggregate set was estimated by applying principal component analysis (PCA) to the concentration levels of the constituent cytokines and using the first principal component (PC) or latent vector score as a measure of joint expression. As a general rule, a second additional principal component was used only if the first component captured less than 80% of the variability in the aggregate set. This was the case for MK1, which was scored as two separate co-expression patterns MK1A and MK1B (Tables 2, 3).

**TABLE 3:** Variance captured by the first principal component (PC1) for aggregated cytokine variables and their respective loadings.

Variable	Aggregated cytokines	Healthy	GWI	Healthy + GWI
MK1a	Tot Variance (PC1)	0.7568	0.8292	0.7873
	IL-1a	0.9877	0.9974	0.9931
	IL-1b	–0.0594	–0.0447	–0.0536
	IL-8	–0.1386	–0.0502	–0.0968
	IL-12	–0.0424	–0.0267	–0.0378
MK1b	Fract. Tot Variance (PC2)	0.1321	0.1075	0.121
	IL-1a	0.1437	0.0513	0.0984
	IL-1b	0.2803	0.3054	0.2972
	IL-8	0.9413	0.9039	0.9267
	IL-12	–0.1218	–0.2950	–0.2078
CK1	Tot Variance (PC1)	0.9234	0.4105	0.8874
	IL-2	0.9917	0.5938	0.9913
	IFN-y	–0.0245	–0.2533	–0.0269
	TNF-a	0.0031	0.7621	0.0149
	TNF-b	0.1261	0.0492	0.1278
CK2	Tot Variance (PC1)	0.9811	0.6788	0.9613
	IL-4	0.0963	0.9225	0.0921
	IL-5	0.7585	–0.0470	0.7568
	IL-13	0.6445	–0.3832	0.6471

*In healthy and GWI models data from respective individual groups was used for PCA whereas all the data was combined in HC + GWI model for PCA. Variance for MK1b represents the fractional variance covered by second principal component (PC2). Data was normalized, interpolated and converted to fold change before the PCA calculations.*

As shown in Table 3, cytokine co-expression patterns were relatively consistent across subject groups for sets MK1A and B, with over 75 and 10% of the overall variability captured by the first and second principal components (PC), respectively. This was not the case for CK1 and CK2 where the shared variability captured by the first principal component PC1 was visibly lower in GWI (0.41 and 0.68, respectively) suggesting that cytokines aggregated under CK1 and CK2 behaved less cohesively in the illness group. With the exception of set CK2, the loading coefficients for PC1 were consistent in sign (positive or negative) across both subject groups. Accordingly, in order to create a common coordinate system and facilitate the comparison of network structures across groups, aggregate expression for these sets was estimated using a PCA model based on profiles from all subjects thereby capturing co-expression patterns shared by both groups (HC and GWI).

#### Creation of Literature-Based Reference Networks

Building on our earlier literature-based model describing cytokine-cell immune signaling (Fritsch et al., 2013), we removed immune cell nodes lying between any two cytokines to create an abstracted graph of cytokine-cytokine interaction. Only edges connecting first (cytokine-cytokine) and second neighbor cytokines (cytokine-cell-cytokine) were included in the final network. In the latter case, the aggregate mode of action linking cytokines was determined by multiplying the sign of the intervening edges. For example, in Fritsch et al. (2013) the MK2 cytokine set inhibits the dendritic cell set (DC1) which typically promotes the secretion of MK15. In the abstraction presented here this translates into a direct inhibition of MK15 by MK2. Also, as MK1 was divided into two subsets, note that all outgoing and incoming edges for MK1 were directly propagated to MK1A and MK1B nodes (Supplementary Figure S2).

To further consolidate this network, we also extracted interactions linking the 16 cytokines measured in our experiments using the “Search Tool for the Retrieval of Interacting Genes” (STRING) database (Szklarczyk et al., 2015). Interactions in the STRING database are included and scored on the basis of several supporting sources such as co-occurrence in manually curated literature and co-expression in available experimental databases to name a few. Every interaction in the STRING database was assigned a confidence score with direction and type of regulation predicted for most edges (except direct physical binding) based on natural language processing (NLP) (Szklarczyk et al., 2015). It is important to note that all the interactions retained in the extracted cytokine network had a confidence score of ≥ 0.80 with respect to their predicted direction based on human studies. Source and target cytokine nodes associated with each edge along with supporting evidence and confidence scores are summarized in Supplementary Table S4. The combined confidence score supporting every association is computed by combining the probabilities from the different evidence channels and corrected for the probability of randomly observing an interaction. For a more detailed description of these scores please see von Mering et al. (2005). We then translated this basic cytokine-cytokine network into one linking the 9 cytokine sets described in Fritsch et al. (2013). Since MK1A, MK1B, CK1 and CK2 individually represent several cytokines in one aggregated variable, multiple edges among constituent cytokines within or between aggregate sets were also rationalized using the union of all incoming and outgoing edges for that cytokine sub-network. This yielded an abstracted network with 38 edges linking 9 aggregate sets from an initial directed network of 47 edges linking 16 individual cytokines extracted from the STRING database. As might be expected these two reference networks overlapped substantially with 30 interactions sharing source, target and direction across both networks. Building on this consensus and including interactions unique to each, we created a single unified reference network with 50 directed edges linking 9-aggregate cytokine nodes (Supplementary Figure S2).

#### Inferring Directed Cytokine Networks From Data

An ordinary differential equation (ODE) based model was used to infer directed interactions among the grouped cytokine variables. These models have been widely used for the inference of regulatory networks. In this model, we use a simple linear rate equation to describe the rate of change of concentration of every aggregated cytokine variable as described in Eq. 1:

(1) $$\frac{\partial x_{i}}{\partial t}=a_{i,1}x_{1}+a_{i,2}x_{2}+\ldots +a_{i,n}x_{n}$$

where *a*_*i,j*_ describes the influence of network node *j* on the rate of change of expression of network node *i*. A positive value of *a*_*i,j*_ represents activation of node *i* by node *j*, negative value represents inhibition and zero value represents no interaction between node *j* and *i*. Eq. 1 can also be rewritten in the matrix form (Eq. 2).

(2) $$\dot{X}\left(t\right)=A\cdot X\left(t\right)$$

where *X* is an *n* × 1 vector and A is an *n* × *n* matrix containing the weight of all edges in the network.

Consistent with our recent work (Vashishtha et al., 2015), we used an extension of standard PCA called partial least squares (PLS) regression (Wold et al., 2001a, b) for the estimation of latent vectors. Furthermore, we used the broken-stick technique, a variant of Horn’s technique (Horn, 1965) to select an appropriate number of latent vectors to be retained for identification of the unknown parameter set *A* in Eq. 2. Note that, this method was chosen over Bartlett’s method (Bartlett, 1950), which is more permissive and therefore more prone to false positives in the inference of interactions (Jackson, 1991; Vashishtha et al., 2015). Parameters of the broken stick method were tuned to provide the maximum F score values for the inference of the combined literature-based reference network described in the previous section (Supplementary Figure S2). A global optimization method, namely constrained simulated annealing, was used to balance computational cost and thoroughness. All algorithms were encoded in MatLab using the functions available in the Statistics, Machine Learning and Global Optimization toolboxes (The MathWorks, Inc., Natick, MA, United States). For more details about parameter tuning we refer the reader to (Vashishtha et al., 2015).

#### Network Analysis

##### Graph edit distance

Weighted Graph edit distances (GED) (Bunke, 2000) were calculated to quantify the topological differences among the networks of same group (intra GEDs) as well as between networks of two groups (inter GEDs). A weighted Graph Edit Distance (GED) corresponds to the “cost” associated with the edit operations to transform one graph into another (Dickinson et al., 2004; Harper et al., 2004). Here, we make the cost of these edit operations proportional to the differences in the edge weights. The weighted *GED*_*A,B*_ between two networks of order N with adjacency matrices *A* and *B* is:

(3) $$GED_{A,B}=\sum_{i=1}^{N}\sum_{j\geq 1}^{N}\left|a_{ij}-b_{ij}\right|$$

where *a*_*i,j*_ and *b*_*i,j*_ are the weights for an element in adjacency matrix *A* and *B*, respectively. Statistical significance of the edit costs separating networks across illness groups compared to networks within groups was based on repeated random sub-sampling of subjects. Local restructuring of the networks that drive these global topological differences was described in terms of node centrality measures such as betweenness centrality, degree centrality and closeness centrality. Furthermore, ‘hubs’ and ‘authority’ centrality scores were used to further differentiate the local topological features of healthy networks from GWI networks. Details of these metrics are described in Supplementary Data Sheet 3. A general review of basic metrics used to describe global and local network structure and their applications in biology may also be found in Barabási and Oltvai (2004) and Huber et al. (2007).

All calculations related to network identification and rationalization as well as the analysis of network attributes were conducted with the MATLAB software environment (The MathWorks Inc., Natick, MA, United States). Note that, MatLab 2016a was used for node centrality measures calculations. The graphical rendering of directed networks was performed using ‘Orthogonal’ layout of yEd graph editor program (yWorks Gmbh, Germany).

##### Simulating network dynamics

The dynamic behavior supported by the directed cytokine networks identified in this work was explored via a discrete state simulation engine NetSim (Di Camillo et al., 2009) where the target transition state for any given cytokine node at time *t* + 1 is determined by resolving the fuzzy logic statement describing the regulation of that node. A sigmoidal activation function is then used by NetSim to modulate the incremental transition from the node’s current state in the direction of its target state. This incremental change in state is weighted by a time constant capturing both synthesis and degradation dynamics. In all simulations the parameters describing node dynamics were sampled from Gaussian distributions with mean and standard deviation as recommended by the authors. As recovery dynamics are of specific interest here, the initial states for each simulation were set to values measured at peak exercise effort.

## Results

Although the exercise capacity of GWI veterans approaches that of healthy controls in terms of time required to reach maximal VO2, they differ significantly in their ability to recover from this challenge. This impaired recovery presents as an exacerbation of GWI symptoms and has been documented in several studies as post-exertional malaise (Cook et al., 2003; Rayhan et al., 2013b). Therefore, we have focused in this work on isolating and comparing the networked response of the immune system in GWI subjects to that of HC subjects in the recovery phase. Specifically, we consider the 4-h time period starting at the VO2 max time point and described by 6 out of the 8 time points measured.

### Divergence in Exercise Response of Individual Cytokines

The distribution of SMETS values describing the separation of time course response in the recovery phase for each individual cytokine both within and between subject groups was calculated using a leave-one-out repeated sub-sampling scheme. The mean values and standard error for intra-group, pooled intra-group (HC + GWI) and inter-group SMETS values describing the separation of the exercise response time courses are reported in Supplementary Table S3A. The corresponding SMETS median values and the median absolute deviation from the median (MADM) are reported in Supplementary Table S3B. Results show that the time course of individual cytokine responses differed significantly between subject groups for most cytokines surveyed based on the Wilcoxon ranksum test and the Student’s t test (Supplementary Table S3C). The SMETS values describing the divergence of responses across groups were significantly different from those computed for subjects within each group separately or when these within-group measures were pooled. The only exceptions were IL-1α, IL-23, TNFα and TNFβ, which did not differ significantly in response course between groups when compared to the separation of responses between healthy control subjects. Of those cytokines that differed most significantly between groups, divergence in IL-2, 13 and 17 responses between GWI from HC subjects produced SMETS values at least 1.5 times those corresponding to the separation of subjects within the same group. It should be noted that in general, cytokine dynamics were more diverse in HC subjects, with higher mean SMETS values separating these subjects in 11 of 16 cytokines. However, only in the case of IL-5, 10, 17, 23 and TNFβ were these within group differences statistically significant.

### Remodeling in Networks of Cytokine Sets Inferred From Experimental Data

In order to align the empirical analysis with the text-mined mechanistically informed model we first projected the cytokine profiles measured at the 6 recovery phase time points into the space defined by the aggregate functional sets described in Tables 2, 3, then captured the response trajectories of these sets by fitting the parameters of a first order linear ODE (Eq. 1). This was performed for each individual subject in each group separately such that subject-specific immune response networks were created that captured the direction (from source to target) and type (activating or inactivating) of interaction linking these functional sets of mediators. To isolate the most robust network features specific to each illness group a combination of leave-one-out cross-validation and bootstrapping was applied as described in the Methods section. We re-sampled 100 random subsets of 10 subjects without replacement from the complete sets of 11 HC and 12 GWI subjects, respectively. Individual cytokine networks were inferred for every subject of each subset. Within each subsample a consensus network was identified by majority rule whereby only those interactions (edges) shared by at least 6 out of the 10 networks were retained. These 100 consensus networks identified for each illness group were used to support comparative statistics describing the significance of network remodeling in GWI (Supplementary Figure S3 and Supplementary Table S5).

**FIGURE 1:**
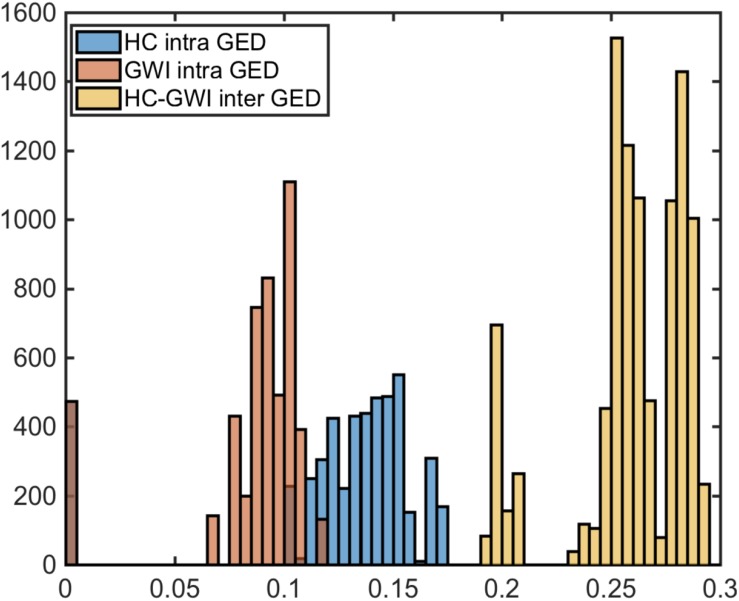
Significantly altered immune circuitry. Graph edit distance (GED) distributions comparison between HC (blue) and GWI (orange) intra GEDs and inter group GEDs (yellow).

Comparing these empirical networks in terms of their overall structure, we found significant remodeling of the topology across groups with the graph edit distance (GED) separating GWI from HC networks significantly exceeding the pooled GED separating networks within the same subject group (Figure 1) (HC and GWI) (*p_inter_* <<< 0.01). In addition, we found that within-group GED was significantly higher in HC compared to GWI (*p_intra_* <<< 0.01) indicating that consensus networks were more topologically diverse in this group. To assess the nature of this remodeling we quantified the role of each cytokine set in the signaling network in terms of centrality measures such as node degree, betweenness centrality and closeness centrality. In addition, hub and authority scores were calculated to further highlight cytokine sets with dominant roles in determining the overall network topology (Supplementary Table S5). Analysis of these measures pointed to a major reshuffling of roles for each cytokine set in the GWI consensus networks. The differences in the median values for the weighted betweenness centrality scores were significant for all cytokine functional sets except MK1B (*p* < 0.05) (Supplementary Table S5A). In general, median scores for the weighted betweenness centrality were higher in HC consensus networks (Supplementary Figure S3) with MK6 (0.25), MK15 (0.21), and CK2 (0.18) nodes being most influential and differing significantly from betweenness values in the corresponding GWI networks (*p* < 0.01) (Supplementary Figure S3 and Supplementary Table S5A). Conversely among the GWI immune networks, cytokine functional sets MK1B (0.20), MK2 (0.16), and MK1A (0.14) exhibited the highest weighted betweenness centrality values, though only in the case of MK2 (*p* < 0.01) did these differ significantly from corresponding values in the HC networks. Recall that a network node with a high betweenness centrality can be thought of as a gatekeeper of information transfer from one segment of the network to another. With this in mind, these analyses suggest that the flow of immune messaging is being directed with less coordinated oversight in GWI and that most of the gatekeepers active in HC have relinquished this role in favor of disproportionately central role for the anti-inflammatory functional set MK2 (IL-10).

This loss of a more centrally mediated oversight of immune signaling in GWI also manifests as broader and more diffuse connectivity between cytokine functional sets in these networks. Indeed, individual cytokine sets could more readily influence (outcloseness centrality), and in turn be more readily affected by other nodes (incloseness centrality) in the GWI immune networks than was the case for HC networks (*p* < 0.05) (Supplementary Figure S3 and Supplementary Table S5B). For example, MK23 (3.74), CK2 (2.75), and MK2 (2.57) were the cytokine functional sets most accessible to the broader immune signaling network (weighted incloseness centralities) in the HC group. In GWI, this greater accessibility was displaced in favor of MK1B (4.38), CK1 (4.35), and MK1A (4.24) in GWI. In terms of breadth of action MK6 (2.96), MK1B (2.67), and MK15 (2.61) displayed the highest median weighted outcloseness in the HC network whereas MK2 (4.25), MK15 (4.11), and MK23 (3.94) functional sets exercised the broadest reach in GWI immune signaling networks. Not surprisingly these differences are in close alignment with median indegree and outdegree values, which were also generally higher in GWI networks (Supplementary Table S5C). With the exception of CK2, all nodes differed significantly in indegree and outdegree between HC and GWI with MK6 undergoing the biggest shift in indegree and MK2 the largest shift in outdegree centrality.

Any shift in connectivity might be expected to have greater significance if it were to disproportionately favor engaging with more influential nodes. Changes in incoming connectivity that result in an increased recruitment of influential source nodes can be quantified as an increase in authority score. Likewise, an increase in outreach to highly influential nodes would translate into an increase in the hub score for that node. Here again we found a broad shift in the patterns of engagement with influential nodes across immune networks in each subject group (Supplementary Table S5D). For example, in the GWI networks identified here, we found that the cytokine functional set CK1 more than doubled in hub score compared to HC, while its authority score decreased by a factor of 4. These measures suggest that the CK1 functional set (IL-2, IFNg, TNFa, and TNFb) increased its regulatory broadcasting and preferentially directed this toward more influential cytokine functional sets despite receiving less prompting by incoming signals from these influential mediators. Similarly, nodes MK1A, MK1B, and MK2 show increased regulatory broadcasting with only modest (fold change (FC) < 2) changes in authority score. Conversely MK15 and MK 23 show markedly lower levels of broadcasting to influential nodes despite similar or higher prompting. Differences across these metrics are summarized in Table 4.

**TABLE 4:**
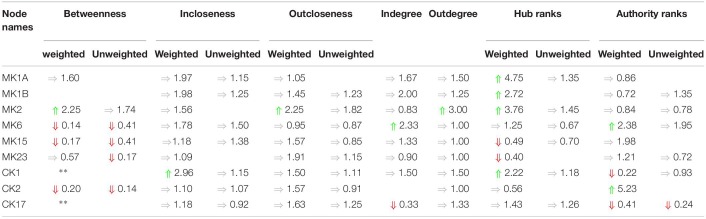
Summary of changes in cytokine node centrality.

*Increase or decrease in fold change for individual node centrality measures (GWI/HC) highlighting p < 0.05 and FC < 0.05 (red down arrow) and FC > 2 (green up arrow), and no significant change (gray arrow). Results show significant change in the roles of a broad majority of aggregate cytokine sets used in the immune signaling model of Fritsch et al. (2013), including MK2, 6 and 15, key components of the GWI regulatory motif. Recall MK1A and B represent the first and second principal components respectively derived from the co-expression patterns of IL-1α, IL-1β, IL-8 and IL-12^∗∗^.*

Collectively these results suggest that the pattern of interactions linking cytokine functional sets has been significantly altered in GWI and that this remodeling has resulted in a re-assignment of the roles these cytokine sets play as information brokers within the immune signaling network. Immune signaling appears more diffuse and less centrally mediated in GWI, with a general trend toward inflammatory functional sets exercising a broader and more influential signaling with less prompting than in needed in HC.

### Concordance With a Literature-Based Reference Network

Based on the subsampling scheme described above, 100 subsamples were created in each of the HC and GWI clinical phenotypes. These 100 subsamples each consisted of 10 subject-specific empirical networks. When casting these against documented signaling mechanisms we found that they were sufficiently diverse in both GWI and HC to collectively canvas over 80% of the documented immune circuitry recruited during exercise in at least half of the instances (median recall 0.82 HC, 0.84 GWI). Similarly, roughly 60% of the regulatory interactions predicted from the data were also documented in the literature in at least half of the cases (median PPV = 0.61 HC, 0.60 GWI) (Supplementary Table S6A). If we restrict this to include only those interactions shared across networks within each subject group, the proportion of documented immune circuitry inferred as active from the data decreases comparably in both HC and GWI to just below 40% (Supplementary Table S6B; median recall 0.37 HC, 0.39 GWI).

**FIGURE 2:**
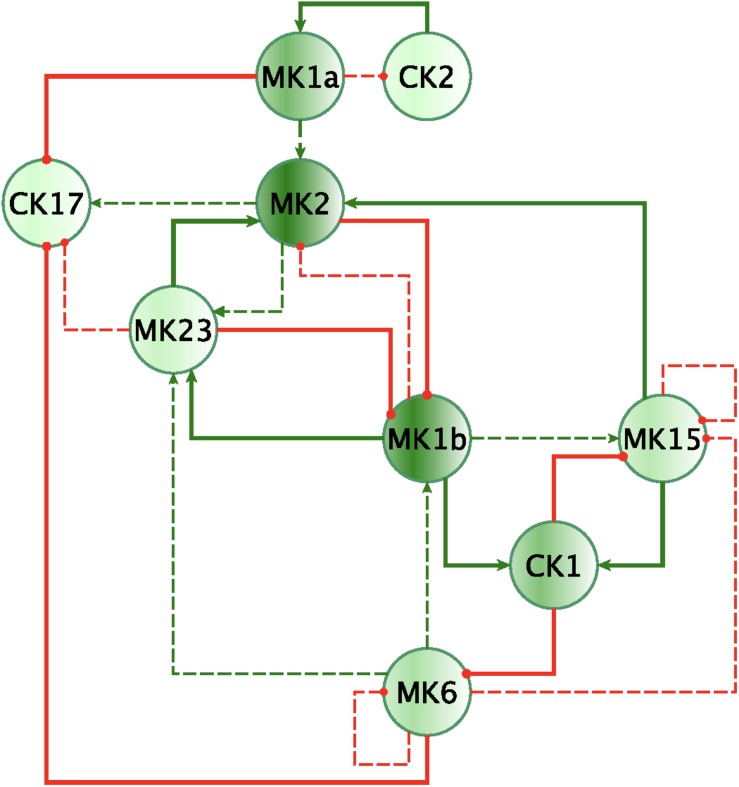
Mechanistically informed GWI regulatory motif. Regulatory interactions extracted from documented prior knowledge that are uniquely represented in the experimental data in GWI during recovery from maximum exercise. Solid lines represent interactions uniquely expressed in HC while dashed lines show interactions shared with GWI. Green arrows indicate a stimulatory action while red “T” terminators indicate suppressive actions.

**FIGURE 3:**
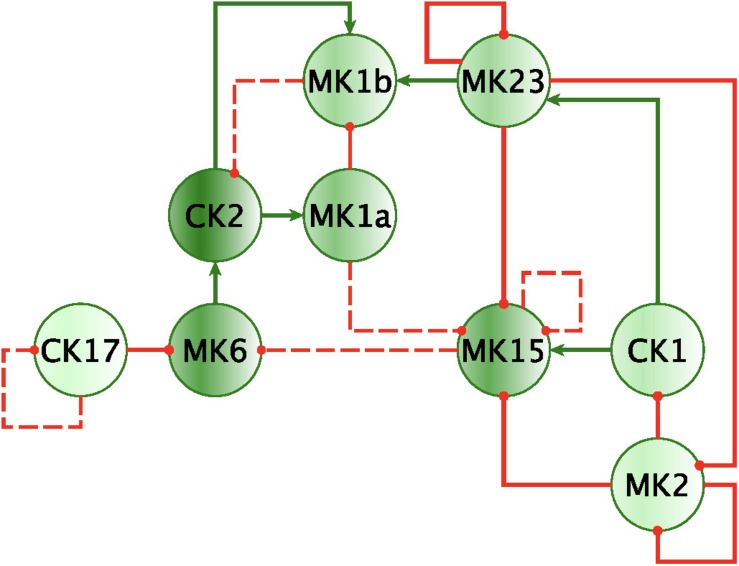
Mechanistically informed HC regulatory motif. Regulatory interactions extracted from documented prior knowledge (STRING and Fritsch et al., 2013) that are uniquely represented in the experimental data in HC during recovery from maximum exercise. Solid lines represent interactions uniquely expressed in HC while dashed lines show control actions shared with GWI. Green arrows indicate a stimulatory action whereas a red “T” terminators indicate suppressive actions.

Finally, by enforcing unanimity across all individual empirical networks assembled from a repeated subsampling time course data in each group, we obtain consensus networks for HC and GWI which converge to a similar number of network interactions (Supplementary Table S6C; 23 HC, 24 GWI) supporting a connection density of roughly 30%. In both HC and GWI groups, network recall, or the proportion of documented interactions inferred as active from the data, decreased almost in step as increasing levels of agreement across individual networks were applied, falling from initial levels > 80% to settle at somewhat similar values of 29% in HC and 22% in GWI. In contrast, positive predictive value (PPV), or the proportion of predicted interactions validated by the literature-based model, increased only slightly in the healthy control group from 61 to 67%, and decreased substantially from 60 to 46% in the GWI group as increased levels of consensus were enforced (Supplementary Table S6C; median PPV 0.67 HC, 0.46 GWI). Notwithstanding spurious false positives expected of this limited group size, this would suggest that in GWI a higher proportion of immune signaling diverges from documented patterns commonly associated with healthy physiology. Focusing our analysis on only those interactions represented unanimously in the data collected in each group and documented in the literature-based model, we obtain conserved characteristic motifs for HC and GWI consisting of 14 and 11 regulatory interactions, respectively (Figures 2, 3 and Table 5). Only 4 of these interactions were shared across illness groups leaving 7 documented regulatory interactions characteristically active in GWI alone. Of these 4 shared interactions, only the positive regulation of MK1A by CK2 retained the same mode of action, the remaining 3 took on opposing regulatory actions across groups suggesting the involvement of unobserved intermediate regulatory elements. In addition, we notice that CK17 is uniquely targeted for active regulation by several mediators in GWI whereas it is an active regulator in the HC circuit. One such regulator of CK17 in GWI is MK23 which is also involved in a characteristic positive feedback loop with MK2 in GWI. Positive feedback regulation is characteristic of self-sustaining cascades and the emergence of multiple stable resting states. In contrast, no positive feedback loops were identified as active during recovery from exercise in HC (Figure 3).

**TABLE 5:**
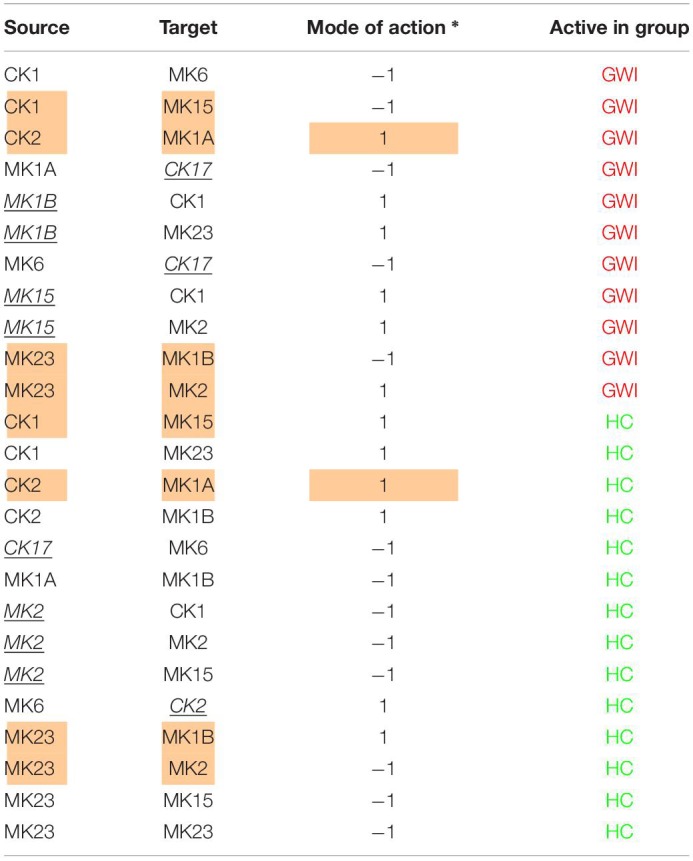
Documented immune signals represented in experimental data.

*Immune signaling interactions documented in the literature (Fritsch et al., 2013) and the STRING database (Szklarczyk et al., 2015) that were also represented in the experimental data collected under exercise challenge in Gulf War Illness (GWI) veterans as well as healthy control veterans (HC). Shaded source-target interactions are shared between both illness groups in direction but not necessarily mode of action. Cytokine sets appearing as unique sources or targets in each group are underlined and italicized. ^∗^Mode of action + 1 is an activator, −1 is an inhibitor.*

**FIGURE 4:**
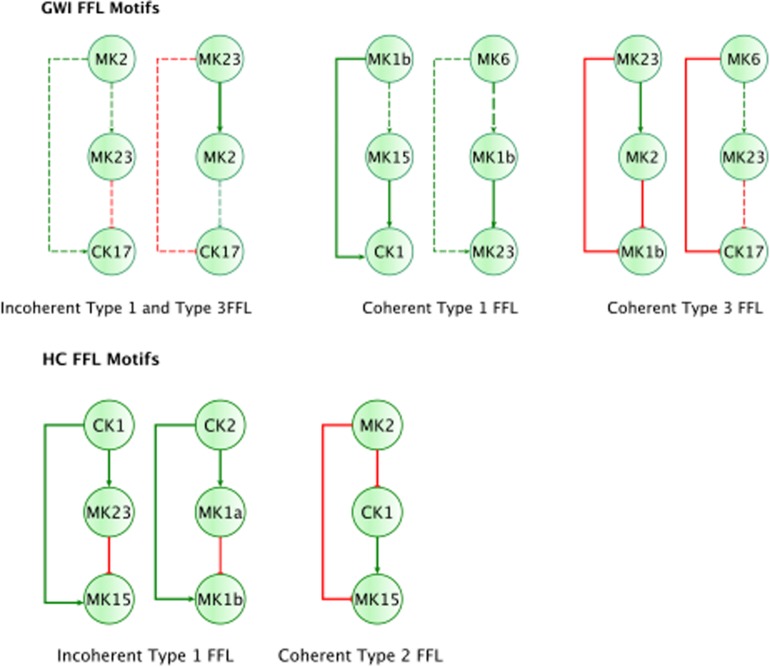
Basic regulatory control motifs in HC and GWI. Minimal regulatory component feedforward control motifs suggested by Alon (2007; Milo et al., 2002) emerge as unique features in the sub-circuits for each illness group. Specifically, coherent feedforward loops (FFL) type 1 and 2 unique to HC (A–C), indicating robustness to sudden disturbances. The GWI circuit however presents with the incoherent type 1 FFL (D), associated with bi-modal behavior.

In addition to feedback regulation, feedforward control circuits are found extensively in nature and serve very specific regulatory functions. Alon (2007) defines 8 such recurring regulatory building blocks or network motifs, classifying them as coherent or incoherent controllers. The latter class of motifs serves as a basic pulse generator and response accelerator whereas the former responds to persistent input with a ‘sign-sensitive delay’, i.e., a controller that delays activation but allows rapid deactivation of a response. Interestingly, the only coherent type 2 motif identified was characteristic of the active HC sub-circuit, suggesting increased robustness to rapid fluctuations in the inflammatory set CK1 and a rapid down-regulation of MK15 by the anti-inflammatory set MK2 (Figure 4). While incoherent type 1 FFL pulse-generating motifs were found active in both groups, the GWI sub-circuit presented with a unique incoherent type 1 regulation of CK17 by MK23. Incoherent feed-forward control motifs produce bimodal behavior (Hart and Alon, 2013) as well as supporting a ratio-based control function, e.g., fold change in signal over background noise (Goentoro et al., 2009). Of note CK17 was also the object of coherent type 3 control in GWI only. In both motifs we find an apparent inhibitory action of MK23 on CK17, which contradicts the well-documented positive contribution of IL-23 to the maintenance and development of Th17 cells (Gaffen et al., 2014). It is important to note that the control action in question was inferred from the experimental data only and was not supported by the literature-based model. Indeed, if we consider the documented control actions in the GWI circuit (Figure 2) we find a cascade linking MK23 to CK17 through the intermediaries MK1B, CK1, and MK6 with alternating control actions such that the net effect is an indirect inhibition of CK17 (Supplementary Figure S4). The incoherent FFL motifs identified should therefore be considered apparent motifs that summarize the net control effects observed. With this in mind, these results suggest an altered and characteristic recruitment of basic regulatory control elements in the oversight of Th17 response to exercise a combined with a reduced engagement in GWI of control elements supporting robust and rapid anti-inflammatory response.

### Simulated ad hoc Cytokine Inhibition

In the previous section, we identified a characteristic control sub-circuitry active during recovery from exercise in each illness group. This active sub-circuitry consists of control actions inferred for experimental data with some of these being further validated against a literature-informed template consisting of known documented mechanistic interactions (Figures 2, 3). One could now ask if there exist minor changes to this circuitry that might allow the GWI sub-circuit to perform at least partially like the healthy control sub-circuit in response to exercise. Mainstream pharmaceutical immune-therapy often involves delivering monoclonal antibodies, cytokine supplementation therapy or small molecules that act as immune receptor agonists or antagonists. As the GWI circuit is *de facto* more abundantly connected we simulate the effects of a pharmaceutical blockade of receptors for a specific cytokine functional group by removing all outgoing edges from that network node. A simulated blockade was applied iteratively to all nodes in the GWI circuit both individually and in pairs with the objective of minimizing the topological differences with the healthy control circuit. The original GWI circuit diverged topologically from the HC circuit with a graph edit distance (GED) of 0.2569 (0.0021 Std. Err.). Results presented in Supplementary Table S7 suggest that attenuating MK6 signaling was the single best intervention target. Blockade of MK6 alone reduced the topological separation of the GWI network from the target HC network to a GED of 0.2427, a small but statistically significant reduction (∼5%). Similarity to the healthy control circuit architecture is further improved slightly by combining antagonism of MK6 action with antagonism of CK1 (GED 0.2338).

**FIGURE 5:**
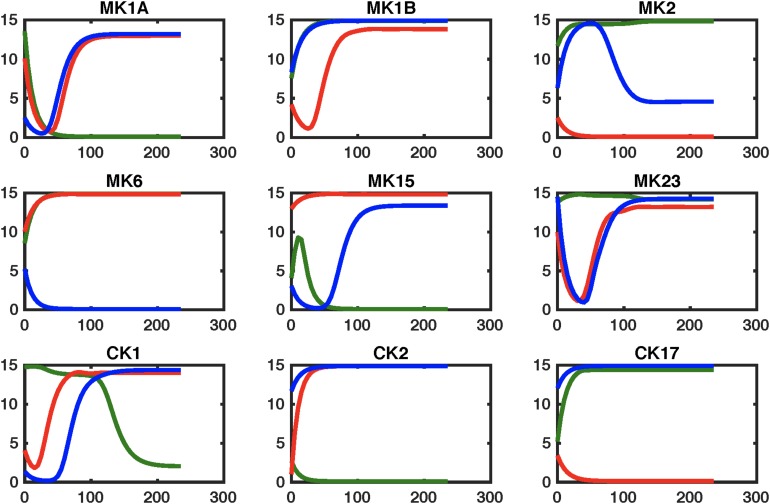
Simulating regulatory circuit response to MK6 and CK1 antagonism. Simulated response to a step perturbation applied to the characteristic circuits for GWI (GWI, red line), healthy control (HC, green line), as well as the pharmaceutically edited GWI network (Treated, blue line). Improved adherence to output from the healthy control circuit are produced for cytokine sets CK17and MK1B, with transient restorative effects on MK2 and MK15. This is accompanied by significant worsening in MK6 response.

**FIGURE 6:**
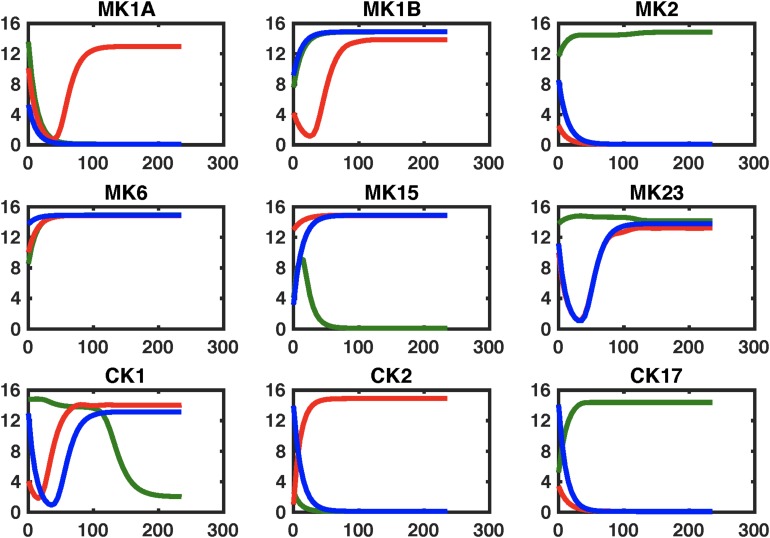
Simulating regulatory circuit response to MK6 and MK23 antagonism. Simulated response to a step perturbation applied to the characteristic circuits for GWI (GWI, red line), healthy control (HC, green line), as well as the pharmaceutically edited GWI network (Treated, blue line). Improved adherence to output from the healthy control circuit are produced for cytokine sets MK1A, MK1B, and CK2 without significant negative effects on other sets.

The effects of these changes in immune network signaling on expected immune response behavior in GWI were then simulated using the NetSim environment in order to provide a qualitative data agnostic perspective. Results presented in Figure 5 suggest that this topologically motivated approach nonetheless produced a rescue of the MK1B and CK17 response, a characteristic component in GWI. Transient restorative effects on MK2 and MK15 responses were also produced but were accompanied by a significant abrogating the otherwise normal MK6 response. The second leading alteration to the GWI immune circuitry consisted in a concurrent blockade of MK6 and MK23 receptor function. Response of this modified GWI immune circuit to a simulated relaxation is described in Figure 6. Results indicate that this strategy would improve adherence to the predicted healthy control circuit response in cytokine sets MK1A, MK1B, and CK2 without inducing significant negative effects in other cytokine responses. It is important to note that these simulations assume that with the exception of pharmaceutically targeted cytokine signals the remainder of the circuitry is still active. Moreover, it also assumes that although some corrective results are produced at the level of individual cytokine sets that the overall immune network continues to operate in the vicinity of the GWI regulatory regime and as such does not significantly activate any new cytokine signaling mechanisms. We have shown previously that certain cytokine sets act as primary drivers of symptom burden, including IL-1a (MK1A) and IL-10 (MK2), and though not curative that mediating these may reduce illness severity (Broderick et al., 2013).

## Discussion

In this study, we used a Graded eXercise Test (GXT) to stimulate immune signaling in a group of veterans with Gulf War Illness (GWI) and matched healthy control subjects. Cytokine levels in blood serum were measured at 8 time points spanning from the initial time point at rest to 4 h post-effort. Statistical analysis using the SMETS metric of the differences in response dynamics for individual cytokines during recovery confirmed that the majority of these differed significantly in GWI compared to HC. The only exceptions to this were responses in IL-1a, IL-23, TNFα, and TNFβ, which did not differ significantly between groups compared to the distribution SMETS distances separating HC subjects from one another. To explore the candidate mechanisms driving these divergent choreographed behaviors we identified directed interaction networks from the data collected in each group, aggregating these cytokines into functional sets to facilitate the integration of these empirical interactions with documented immune signals described in work by Folcik et al. (2007, 2011) and used subsequently in simulations by our group (Fritsch et al., 2013). This literature-based network was further reinforced by including immune signaling interactions reported in the latest version of the STRING database (Szklarczyk et al., 2015). Integration of literature-based and data-derived signaling components indicated that vastly different subsets of immune circuitry become active in each group during recovery with a smaller proportion of these being documented in our reference circuit for GWI than HC. Significant differences in topology occurred specifically regarding involvement in the broader network of MK2, MK6, and MK15, key components of the GWI regulatory motif. This remodeling of the signaling network manifested in part through the emergence of characteristic feedforward control motifs proposed as basic regulatory building blocks by Alon (2007). Not surprisingly feedforward mediation of MK23 and CK17 by MK2 and MK6 emerged as distinguishing control elements that were characteristically active in GWI during recovery from exercise. Interestingly, when assessing topological changes that might be imparted to the GWI sub-circuit to produce a circuitry that best resembles that of HC, abrogating MK6 (IL-6) signaling arose as the single most impactful modification. Combining this with a concurrent blockade of an inflammatory Th1 cytokine under CK1 (IL-2, IFNg, TNFa, and TNFb) further improved alignment in topology between the GWI and HC active sub-circuits. Simulating the effects of these changes in connectivity suggested that this joint blockade of MK6 and CK1 might allow for the recovery of normal response dynamics in MK1B (primarily IL-1b and IL-8), MK2 (IL-10), and CK17 (IL-17) response dynamics albeit while worsening MK6 (IL-6) response. In previous work by our group (Broderick et al., 2013) IL-10 (MK2) arose as a strong correlate of increased illness severity, specifically multidimensional fatigue inventory (MFI) (Smets et al., 1995) describing increased general and physical fatigue accompanied by reduced activity and motivation scores. Increases in IL-10 also correlated with decreased general well-being scores under the SF-36, a 36-item short-form survey (Ware and Sherbourne, 1992) assessing health-related quality of life. In addition to reducing symptom burden, theoretical simulations by our group (Craddock et al., 2015) identified inhibition of Th1 (CK1) inflammatory cytokines as a main component in a two-pronged intervention that could potentially deliver lasting remission from GWI.

The pharmaceutical blockade strategy that delivered the next best topological alignment of GWI and HC sub-circuits consisted of jointly inhibiting MK6 (IL-6) and MK23 (IL-23) receptors. Interestingly IL-23 modulation has recently attracted interest as a potentially important therapeutic target relevant to a broad range of autoimmune illnesses (Gaffen et al., 2014; Yang et al., 2014; Sasaki-Iwaoka et al., 2018). Moreover, activation of STAT3, a key component of IL-23/IL-17 signaling has been linked to neurotoxin induced neuro-inflammatory hyper-responsiveness in a mouse model of GWI (Locker et al., 2017). Similarly, selective antagonism of IL-6 receptor signaling has shown established efficacy in treating several autoimmune illnesses and in repairing Th17/Treg imbalance (Tanaka et al., 2012). Moreover, anti-IL-6 therapies have proven especially useful for example in treating rheumatoid arthritis in patients unresponsive to TNF inhibitors (Tanaka and Martin Mola, 2014). Simulation of this dual IL-6/IL-23 blockade suggests that this strategy might support the rescue of both MK1A and MK1B (IL-1a, IL-1b, IL-8, and IL-12) as well as responses in Th2 cytokines under CK2 (IL-4, 5, and 13). This is accomplished under this scenario without negatively impacting other responses. Once again, our previous work indicated that changes in IL-4 and IL-12 correlated significantly with changes in MFI scores for motivation and the Krupp Fatigue Severity Inventory (Krupp FSI) (Krupp et al., 1989). Likewise changes in IL-1a and IL-5 correlated with changes in SF36 measures for physical function, physical limit, pain, and vitality. Moreover, results of another analysis by our group (Broderick et al., 2011) suggested that initial variations in IL-1a levels may catalyze much broader immune activation during exercise and serve as an important driver of exacerbation in GWI.

Collectively these results suggest that pharmacologically altering characteristically active elements of the immune circuitry can inform on strategies that while not curative may nonetheless deliver reduction in symptom burden in GWI. It is important to recall that these characteristic circuits represent immune signaling that is predominantly active within the stable regulatory regime that we propose perpetuates GWI (Craddock et al., 2014). As such, we propose that while a broader response circuitry is available in principle, for the most part the topology of this active sub-circuitry remains dominant within this homeostatic regime and these pharmaceutical interventions are reasonably well represented by edits to this characteristic circuit. This assumption becomes less valid the greater the deviation from the chronic stable state. Moreover, it is also important to remember that GWI is known for its heterogeneity with presentation and severity being associated with area of deployment in theater and the corresponding exposure profile (Steele et al., 2012). Though the longitudinal sampling used here represents a significant experimental effort even at this limited group size, the assumption remains that these GWI subjects are reasonably representative of the broader illness population. Indeed, a core premise for this work was that interactions between immune markers might be conserved more robustly across individuals than changes in marker expression as they may better reflect core illness mechanisms.

It should also be noted that the current work remains a coarse-grained description of immune signaling and that the interactions inferred from the data and extracted literature are the aggregate end result of a myriad of intracellular signaling events. Further informing on the validity of these interactions by enforcing formal compliance with known pathway level processes which have been more directly validated would contribute added rigor and should be pursued. For example, intracellular transcriptional signaling networks that govern the behavior of key cell populations such as NK cells (Liquitaya-Montiel and Mendoza, 2018), T lymphocytes (Martínez-Sosa and Mendoza, 2013) and others might be introduced as separate model compartments and these subnetworks connected explicitly through soluble mediators as in Mendoza (2013). Ideally one would also have available at least partial transcriptomic data to inform or at least partially validate such a model. Certainly, this would deliver a much higher resolution description of immune dysfunction in GWI. Though data supporting this level of resolution was unavailable in this study, we propose that the approach presented here nonetheless offers a simple framework for introducing documented functional relationships into an otherwise naïve mining of experimental data describing soluble immune mediator expression in GWI. Accordingly, at least in broad functional terms, the resulting functional motifs may more reliably inform on beneficial strategies for illness management than would purely conventional statistical network identification.

## Data Availability Statement

The raw data supporting the conclusions of this manuscript will be made available by the authors, without undue reservation, to any qualified researcher. Summary statistics describing the data are reported in this work in Supplementary Table S1.

## Ethics Statement

All subjects signed an informed consent approved by the Institutional Review Board of the University of Miami and the Miami Veterans Affairs Medical Center. Ethics review and approval for data analysis was also obtained by the IRB of the University of Alberta. The work reported in this manuscript consists of a secondary analysis of existing data.

## Author Contributions

SV developed and evaluated the mathematical analysis tools, conducted the analyses, prepared graphics and drafted the initial manuscript. GB oversaw the design of the mathematical tools and the analyses and co-wrote the initial manuscript. TC reviewed the design of the methods, consulted on the methodology, co-wrote and edited the manuscript. ZB oversaw the collection, filtering and normalization of the raw data. FC and EB oversaw study coordination, recruitment and processing of all subjects. MF designed the study, oversaw all laboratory assessments, sample collection and processing as well as contributing directly to the interpretation of the results. NK designed the study, directed all clinical and scientific aspects of the overall study contributing directly to the study design and the interpretation of results. All authors have read and approved the final manuscript.

## Conflict of Interest

The authors declare that the research was conducted in the absence of any commercial or financial relationships that could be construed as a potential conflict of interest.
