# Supplementary material for: Leveraging Prior Knowledge to Recover Characteristic Immune Regulatory Motifs in Gulf War Illness
Source: Front Physiol. 2020 Apr 28;11:358. doi: 10.3389/fphys.2020.00358 (PMC7198798; doi:10.3389/fphys.2020.00358)
Supplement: Supplementary file 2 [file Data_Sheet_2.DOCX]

Supplementary Material

Supplemental Figures S1-S4

Saurabh Vashishtha^1, 2^, Gordon Broderick^2,3^, Travis J. A. Craddock^4,5^, Zachary M Barnes^6,7^, Fanny Collado^7^, Elizabeth G Balbin^4^, Mary Ann Fletcher^4,5,7^, Nancy G Klimas^4,5,7^

^1^Dept. of Medicine, University of Alberta, Edmonton, AB, Canada

^2^Center for Clinical Systems Biology, Rochester General Hospital, Rochester, NY, USA

^3^Dept. of Biomedical Engineering, Kate Gleason College of Engineering, Rochester Institute of Technology, Rochester, NY, USA

^4^Inst. for Neuro-immune Medicine, Nova Southeastern Univ., Ft Lauderdale, FL, USA

^5^Depts. of Psychology & Neuroscience, Computer Science, & Clinical Immunology, Nova Southeastern Univ., Ft. Lauderdale, FL, USA

^6^Diabetes Research Institute, University of Miami, Miami, FL, USA

^7^Miami Veterans Affairs Medical Center, Miami, FL, USA

*** Correspondence:**Gordon Broderick, PhD, Director

Center for Clinical Systems Biology, Rochester General Hospital

1425 Portland Avenue

Rochester, NY 14621
gordon.broderick@rochesterregional.org

**Figure S1. Overview of cytokine expression**. Fold change in cytokine expression at each time point for individual healthy control subjects (HC) over average levels for the healthy control group.

**Figure S2. Literature based aggregated cytokine network.** Cytokine network extracted from the immune signaling network in Fritsch et al. 2013 combined with cytokine network extracted from the STRING database. Green edges terminating in an arrow represent activation of the target node by the source node whereas red edges terminating in a T shape represent inhibition of the same. Edges with a thick solid line represent a shared interaction fond both in the model of Fritsch et al. (2013) based on the Basic Immune Simulator (BIS), and the network reported in the STRING database (Szklarczyk et al. 2015) abstracted into the same composite cytokine sets.

**References**.

Fritsch, P., Craddock, T.J., del Rosario, R.M., Rice, M.A., Smylie, A., Folcik, V.A., de Vries, G., Fletcher, M.A., Klimas, N.G., and Broderick, G. (2013). Succumbing to the laws of attraction: Exploring the sometimes pathogenic versatility of discrete immune logic. *Sys. Biomed*., 1(3):1. doi: 10.4161/sysb.28948.

Szklarczyk, D., Franceschini, A., Wyder, S., Forslund, K., Heller, D., Huerta-Cepas, J., Simonovic, M., Roth, A., Santos, A., Tsafou, K.P., Kuhn, M., Bork, P., Jensen, L.J., and von Mering, C. (2015). STRING v10: protein-protein interaction networks, integrated over the tree of life. *Nucleic Acids Res.*, 43(Database issue): D447-452. doi: 10.1093/nar/gku1003.

**Figure S3. Comparison of weighted node centrality measures.** Weighted betweenness, incloseness, outcloseness, hub and authority centrality scores of healthy consensus networks (Black boxes) were compared with their counterparts in GWI consensus networks (red boxes). Lines inside the boxplots show the median values and red (+) signs show outliers.

**Figure S4: Expanded inferred association of MK23 with CK17**. Deconstruction of overall net inhibition of CK17 by MK 23 inferred from the data (dashed lines) into the cumulative indirect component control actions documented in the literature (solid lines) (green edges activate; red edges inhibit). The apparent inconsistency with reported stimulation of the Th17 axis by IL-23 consists of indirect effects involving mediation unmeasured immune elements (grey nodes), specifically cortisol and dendritic cell (DC) abundance.
