# Supplementary material for: Leveraging Prior Knowledge to Recover Characteristic Immune Regulatory Motifs in Gulf War Illness
Source: Front Physiol. 2020 Apr 28;11:358. doi: 10.3389/fphys.2020.00358 (PMC7198798; doi:10.3389/fphys.2020.00358)
Supplement: Supplementary file 3 [file Data_Sheet_3.DOCX]

Supplementary Material

Details of Network Topology Metrics

Saurabh Vashishtha^1, 2^, Gordon Broderick^2,3^, Travis J. A. Craddock^4,5^, Zachary M Barnes^6,7^, Fanny Collado^7^, Elizabeth G Balbin^4^, Mary Ann Fletcher^4,5,7^, Nancy G Klimas^4,5,7^

^1^Dept. of Medicine, University of Alberta, Edmonton, AB, Canada

^2^Center for Clinical Systems Biology, Rochester General Hospital, Rochester, NY, USA

^3^Dept. of Biomedical Engineering, Kate Gleason College of Engineering, Rochester Institute of Technology, Rochester, NY, USA

^4^Inst. for Neuro-immune Medicine, Nova Southeastern Univ., Ft Lauderdale, FL, USA

^5^Depts. of Psychology & Neuroscience, Computer Science, & Clinical Immunology, Nova Southeastern Univ., Ft. Lauderdale, FL, USA

^6^Diabetes Research Institute, University of Miami, Miami, FL, USA

^7^Miami Veterans Affairs Medical Center, Miami, FL, USA

*** Correspondence:**Gordon Broderick, PhD, Director

Center for Clinical Systems Biology, Rochester General Hospital

1425 Portland Avenue

Rochester, NY 14621
gordon.broderick@rochesterregional.org

In this work the following graph theoretic measures were defined as follows:

1. ***Node degree centrality***. The node degree of a node *n* is the number of edges linked to *n*. A directed network has two categories of node degree namely *outdegree* and *indegree.* The number of edges directed outwards from a node is counted as the outdegree whereas number of edges directed towards a node is counted as the indegree of that node.
2. ***Node betweenness centrality***. The node betweenness centrality $C_{b}\left( n \right)$for each node *n* of a network was calculated using the Brandes algorithm (Brandes, 2001). The betweenness centrality of a node *n* reflects the amount of control that this node exerts over the interaction between communities of neighboring nodes in the network (Yoon et al., 2006) and can be computed as follows:

$C_{b}\left( n \right)=\sum_{s\neq t\neq n} \left( \frac{\sigma_{s,t}(n)}{\sigma_{s,t}} \right)$ (1)

where *s* and *t* are source and target nodes in the network different from *n*, $\sigma_{s,t}$ denotes the number of shortest paths from all *s* to all *t*, and $\sigma_{s,t}(n)$ is the number of all shortest paths from s to *t* that must pass through node *n*. In this work, both weighted and unweighted betweenness centralities were calculated for each node of every network. Note that, weighted and unweighted betweenness centrality scores were normalized for every node as ${C_{b}(n)}/\left( (N-1)(N-2) \right)$

1. ***Closeness centrality***. Closeness centrality *C_c_(n)*of node *n* is the inverse sum of the shortest path length from a node to all other nodes of the network and represents the importance of a node in context of information processing. It is a measure of how fast information spreads from a given node to other reachable nodes in the network (Newman, 2003) and can be calculated as follows:

$C_{c}\left( n \right)=\left( \frac{A_{n}}{N-1} \right)^{2}\frac{1}{C_{n}}$ (2)

where *A_n_* is the number of reachable nodes from node *n*, *N* is the number of nodes in the network and *C_n_* is the sum of path lengths from node *n* to all reachable nodes. In directed networks, closeness is represented as incloseness and outcloseness. Unlike outcloseness, incloseness is a measure of path lengths from all other nodes to a node *n*. Weighted and unweighted in and out closeness centrality scores were calculated for each network.

1. ***Hubs and authorities***. The Hubs and Authorities centrality scores are two linked centrality measures that are recursive. Klienberg’s HITS algorithm (Klienberg, 1999) was used to iteratively calculate weighted and unweighted scores. The Hubs score of a node is the sum of the Authorities scores of all its successors. Similarly, the Authorities score is the sum of the Hubs scores of all its predecessors. Both scores are normalized such that the sum of all Hubs scores as well as sum of all Authorities scores is 1. As a result, nodes that receive high Hub scores typically have broadly distributed outgoing traffic whereas nodes with high Authority scores typically show broad incoming traffic.

**References**.

Brandes, U. (2001). A Faster Algorithm for Betweenness Centrality. *J. Math. Sociol*., 25(2):163-177. doi: 10.1080/0022250X.2001.9990249

Kleinberg, J. (1999). Authoritative sources in a hyperlinked environment. J. ACM., 46 (5): 604–632. doi: 10.1145/324133.324140

Newman, M.E.J. (2003). The Structure and Function of Complex Networks. *SIAM Rev*., 45(2): 167–256. doi: 10.1137/S003614450342480

Yoon, J., Blumer, A., Lee, K. (2006). An algorithm for modularity analysis of directed and weighted biological networks based on edge-betweenness centrality. *Bioinformatics*. 22(24): 3106-3108. doi: 10.1093/bioinformatics/btl533
